# Emergency Response Measures for Anesthesia Nursing During the COVID-19 Pandemic: West China Hospital Experiences

**DOI:** 10.3389/fmed.2020.00460

**Published:** 2020-07-22

**Authors:** Ping Zheng, Ruihao Zhou, Lu Yin, Xiaorong Yin, Yongqiao Mao, Heng Wang, Ling Ye, Tao Zhu

**Affiliations:** ^1^Department of Anesthesiology, West China Hospital, Sichuan University, Chengdu, China; ^2^West China School of Nursing, Sichuan University, Chengdu, China; ^3^Department of Pain Management, West China Hospital, Sichuan University, Chengdu, China

**Keywords:** novel coronavirus pneumonia, COVID-19, epidemic prevention, anesthesia nursing, nursing management

## Abstract

During the COVID-19 pandemic, ensuring the gradual recovery of anesthesia nursing unit and avoiding cross-infection between surgical patients and staff are difficult problems for hospital managers. We outlined the emergency response measures and the transition to normal operation of the anesthesia nursing unit in West China Hospital, which is a large teaching hospital. This mainly included hospital and operating room channel management, three-level screening management of patients and medical staff, classification management of patients undergoing anesthesia and recovery, training management of medical personnel, strict environmental management, and online teaching management.

## Introduction

A novel coronavirus pneumonia outbreak occurred in Wuhan, Hubei, China, in December 2019 (1, 2). Following more than 2 months of struggle, China's epidemic ultimately began to show a downward trend (3). During the coronavirus disease (COVID-19) pandemic, ensuring the gradual recovery of anesthesia nursing unit and avoiding cross-infection between surgical patients and staff are difficult problems for hospital managers. West China Hospital of Sichuan University, a comprehensive teaching hospital with more than 4,000 beds, is an emergency and critical treatment center in Western China. To effectively meet the needs of daily diagnosis and treatment, from February 10, 2020, the anesthesia nursing unit, as well as the operation of elective surgery, was slowly resumed. According to the operation in the past month, we outlined the epidemic prevention and control strategies for anesthesia nursing units and then strictly implemented them.

From February 10 to March 20, 2020, 2213 patients underwent postoperative anesthesia recovery nursing. No cross-infection occurred in the COVID-19 hospital, and no adverse nursing events took place. During the epidemic period, it was impossible to stop all scheduled operations. Our aim is to help anesthesia and nursing departments worldwide based on our experience of epidemic prevention and control, as will be discussed in the following text.

**Figure 1:**
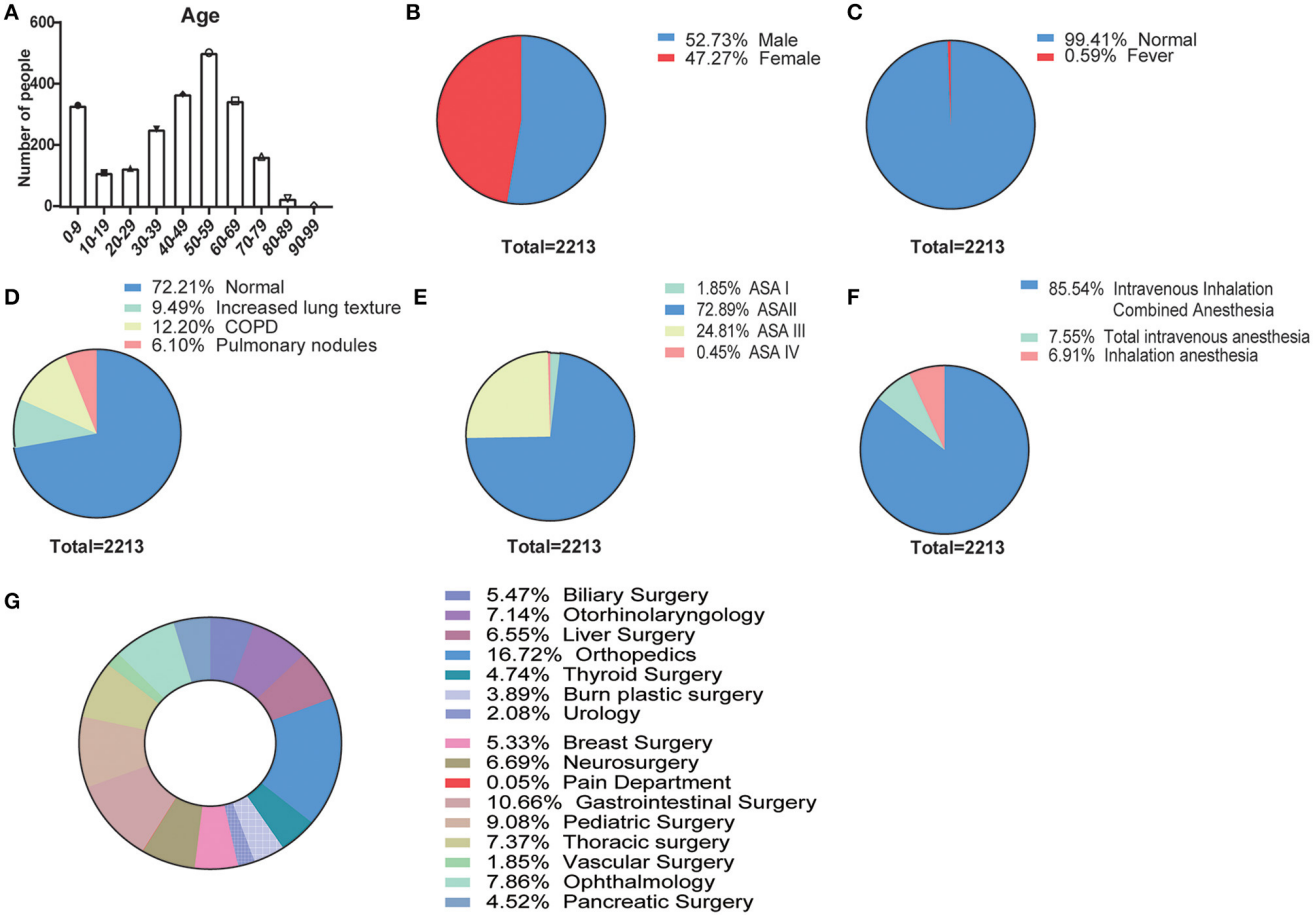
Baseline characteristics of patients who underwent postoperative anesthesia in the COVID-19 pandemic (*N* = 2,213). **(A)** Age frequency distribution of the patients for every 10 years; pie charts of **(B)** gender distribution (male, female), **(C)** preoperative fever (≥37.3°C, normal, fever), **(D)** 3-day preoperative CT (normal, increased lung texture, COPD, pulmonary nodules), **(E)** ASA grade (ASA I, ASA II, ASA III, and ASA IV), **(F)** anesthesia methods (intravenous inhalation combined anesthesia, total intravenous anesthesia, and inhalation anesthesia), and **(G)** surgical types.

We statistically evaluated the basic information of the 2,213 patients, such as age, gender, preoperative fever, fever patients with COVID-19 nucleic acid test, preoperative CT, ASA grade, anesthesia method, and operation type. The age frequency distribution of the patients was mainly distributed in the following age groups: 50–59, 40–49, 60–69, and 0–9 years old (Figure 1A). Also, the gender distribution (Figure 1B) was 1,167 males (52.7%) and 1,046 females (47.3%). The patients were also assessed for preoperative fever (≥37.3°C), and the nucleic acid test results of 13 patients (0.59%) with fever (Figure 1C) were negative. The principal distribution (Figure 1D) of patients with 3 days preoperative CT was normal (*n* = 1,598, 72.2%), and increased lung texture (*n* = 210, 9.5%), COPD (*n* = 270, 12.3%), and Pulmonary nodules (*n* = 135, 6.1%) were noted. The ASA grade (Figure 1E) was chiefly composed of ASA I (*n* = 41, 1.9%), ASA II (*n* = 1,613, 72.9%), ASA (*n* = 549, 24.8%), and ASA IV (*n* = 10, 0.5%), and anesthesia methods (Figure 1F) were principally intravenous inhalation combined anesthesia (*n* = 1,893, 85.54%), total intravenous anesthesia (*n* = 167, 7.55%), and inhalation anesthesia (*n* = 153, 6.91%); in addition, the top 3 surgical types (Figure 1G) were orthopedic surgery (*n* = 370), gastrointestinal surgery (*n* = 236), and pediatric surgery (*n* = 201).

## Hospital and Operating Room Channel Management

### Implementation of Three-Channel Management for Patients in All Buildings

**Figure 2:**
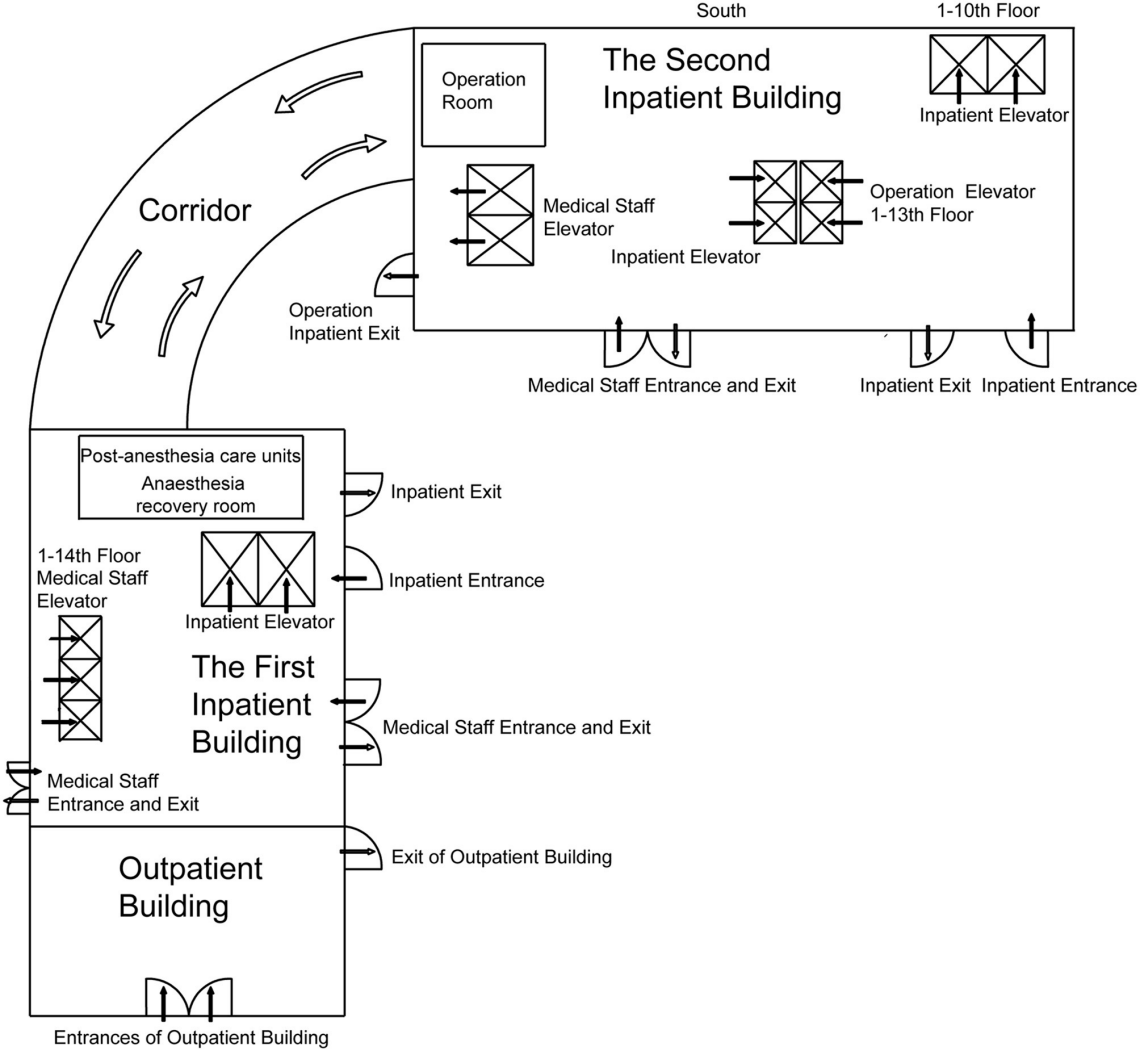
The building plan of the outpatient building and inpatient buildings 1 and 2. The post-anesthesia care units are on the 10th floor of the first inpatient building, and the operating rooms are on the 11th to 13th floor of the second inpatient building. The two buildings are connected by corridors.

We report the building plan and channel management of the outpatient building, the first inpatient building, and the second inpatient building of West China Hospital (Figure 2). According to the hospital outpatient spatial structure, the patient treatment route was converted to one-way, and the entrances and exits were, respectively, arranged at the two ends of the outpatient building. Patients could only enter from the entrance and exit from the exit, and the rest of the access was closed in the meantime.

The implementation of “three-channel” management in all buildings of the hospital meant that the entrances and exits of operation inpatients, inpatients and their escorts, and medical staff were separated and run separately without crossing each other (Figure 2). Patients and the person accompanying them were given an admission certificate. Personnel with no certificate were not allowed to enter under strict control. All personnel entering hospital buildings had to wear masks and take temperature measurements properly.

### Transformation of Patient Access to the Operating Room

The route of patients entering and leaving the operating room was fixed to one-way transport. Prior to the operation, their temperature was taken thrice in the entrance of the inpatient buildings and operating rooms and also inside the operating rooms. When the temperature was not normal, a report had to be made to the infection management department of the hospital that included the patient's epidemiological history. Experts from the infectious disease department and respiratory department would determine whether the operation could be pushed through as usual following consultation. Following the operation, the patient's tracheal intubation or laryngeal mask had to be removed in the operating room, and then the patients were transferred to the anesthesia recovery room for recuperation. The post-anesthesia care units were on the 10th floor of the first inpatient building, and the operating rooms were on the 11th to 13th floor of the second inpatient building. The two buildings are connected by corridors. If the vital signs of patients were stable and reached the standard of withdrawal from the anesthesia recovery room, the anesthesia nurse would return them to the ward via the “operation inpatient elevator.”

## Three-Level Screening Management of Patients and Medical Staff

### Surgical Patients During Hospitalization

In theory, patients were not allowed to come with an accompanying person. However, in special cases, “one patient, one accompanying person” (one fixed person) was strictly implemented. The head nurse would evaluate the epidemiological history and then issue the accompanying certificate.

A “three-level screening” system was applied in each ward and outlined as follows:

1. First-level screening: Each nursing unit was under the control of a specially assigned person for 24 h, and the patient or/and the accompanying person had to enter with a certificate. The temperature and epidemiological history had to be inquired about and registered.
2. Second-level screening: The patient's temperature had to be monitored as required, and the temperature of the accompanying person had to be monitored thrice a day. The epidemiological history, dizziness, chest tightness, fatigue, and other symptoms had to be inquired about so that people having these problems could be found in time and reported to the infection management department of the hospital.
3. Third-level screening: When the doctor did rounds in the room, the patient would again be asked about his/her epidemiological history and whether he/she had dizziness, chest tightness, and fatigue. Patients had to complete a chest CT examination prior to the operation to check whether they had COVID-19 (4).

### Pre-job Epidemiological Screening of Medical Staff

Following the Chinese Lunar New Year holiday, the medical staff needed to report to the department managers their health status, their activity regarding going out and returning during the epidemic, their epidemiological contact history, etc. Before returning to work, those who had left Sichuan Province and returned to Chengdu for <14 days had to isolate at home and not return to work. Until the isolation time reached 14 days following return with no infection symptoms, they needed to apply to the department for a certificate to return to work. Medical staff had to wear masks and have work permits, and a special passage was set up for them before entering and leaving each inpatient building. Temperature measurement and registration were again performed before lunch and on leaving the department after work.

## Classification Management of Patients Undergoing Anesthesia and Recovery

### Special Management of Ordinary Patients

Following the operation, the tracheal intubation or laryngeal mask was removed from the patient in the operating room, and they were then sent to the anesthesia recovery room for further assistance. Not only could this save on medical protective materials, but it also avoided the spread of aerosol in the recovery room due to tracheal intubation and extubation. Following the extubation of the tracheal intubation or laryngeal mask, patients were given low-flow oxygen via a facemask with a reservoir and a medical surgical mask once they were breathing smoothly and were then sent to the anesthesia recovery room.

At the end of each operation, the operating room would be disinfected. In addition to routine anesthesia and care, special attention was paid to ensuring the following:

1. The space between beds of resuscitated patients was >1 meter to lessen the possibility of cross-infection between patients.
2. In the recovery room, patients used a low-flow, non-humidification nasal prong to inhale oxygen, reducing aerosol production.
3. During the recovery period, the patients wore masks and were given low-flow nasal oxygen.
4. The patient's temperature was monitored. If the temperature exceeded 37.3°C, it was reported immediately to the head nurse, the surgeon in charge, and the anesthesiologist during the operation. The causes of fever were assessed in different ways until the possibility of infection with COVID-19 was eliminated.
5. The use of antiemetics could lessen postoperative nausea, vomiting, and the possibility of exposure.
6. When patients with tracheotomy needed sputum suction, medical staff had to wear anti-seepage isolation clothing and goggles or a face screen on the basis of standard prevention (5).
7. After the patient left the anesthesia recovery room, the used instruments, surface, bed unit, cardiac conductivity line, blood pressure cuff, blood oxygen saturation fingertip, etc. were wiped with a disinfectant containing 500 mg/L chlorine, and the humidification bottle was placed into a white garbage bag with a cover.
8. When transferring patients back to the ward, the shortest route and the special elevator for surgical patients were taken.
9. The disposable bedspread and quilt cover were replaced by one person only, and the part of the transfer vehicle that had come into contact with the patient was wiped with a disinfectant containing 500 mg/L chlorine.

### Special Management of Suspected/Confirmed Patients

In theory, suspected/confirmed patients should have no elective surgery, as the impact of surgery may aggravate the patient's condition or even lead to the death of the patient. Only emergency rescue surgery was performed (6). The operation and postoperative recovery were completed in a negative-pressure operating room.

Aside from the routine recovery nursing, the following should be paid attention to when patients are being intubated and recovering in the negative-pressure operating room (7):

1. The air pollution in the operating room should be lessened as much as possible, a breathing filter at the connection between the patient's tracheal intubation and the threaded pipe of the anesthesia machine should be installed, and a breathing filter between the suction and the exhalation of the anesthesia machine should be installed as well.
2. Under deep anesthesia, secretion in the airway should be suctioned to lessen the incidence of cough.
3. There should be someone to assist the anesthesiologist in the removal of the tracheal intubation when the patient's spontaneous breathing pattern has not recovered.
4. When the tracheal tube is pulled out, the breathing filter at the end of the tube should be kept and connected with the breathing mask, which should be tightly connected with the patient's nose and mouth and connected with the anesthetic breathing circuit.
5. Following the stoppage of oxygen inhalation, the patient should wear a medical protective mask right away.
6. The prophylactic use of antiemetic drugs to avoid nausea and vomiting complications should be noted.
7. Once the patient is deemed ready for discharge, the infection management department and the medical department of the hospital should be asked to work out the transfer route and make preparations for the transfer route and docking with the isolation ward.
8. The transport personnel ought to wear protective equipment in accordance with the level three protection standard, carry a sealed special transport rescue box (including the breathing bag, breathing mask, sputum suction tube, and 50-ml syringe), and use a negative-pressure transfer bed to transport the patients back to the isolation ward.
9. The treatment of articles, instruments, and equipment used by patients and the environmental treatment of the operating room and buffer room should be performed in accordance with the regulations on the management of medical wastes, the technical specifications for disinfection of medical institutions, the management specifications for hospital air purification, and the management specifications for environmental surface cleaning and disinfection of medical institutions.
10. The medical staff in the operating room should only leave the negative-pressure operating room after taking off their medical protective equipment in accordance with the standard process and hand hygiene.

## Training Management of Medical Personnel

### Training on the Front Line

The hospital adopted the forms of live broadcast, a TV morning meeting, WeChat enterprise number push, and so on, to report COVID-19-related knowledge to medical staff, students, and logistics personnel in all hospitals and to train the personnel on how to wear protective equipment and relieve mental stress (8). At the end of each course, an online theoretical assessment was performed to complete the first step of advanced training.

### Offline Training After Arrival

The selection of personal protective equipment (PPE) in place of standard and corresponding protective measures and various treatment activities in clean areas, potential pollution areas, and pollution areas was made clear. The aim was that protection of medical personnel would be implemented, but overprotection would be ended. When nurses assist in tracheal intubation, sputum suction, tracheal intubation extubation, fiberoptic bronchoscopy, etc., which may generate splashes or aerosols, they should wear medical protective masks, goggles/protective screens, and anti-seepage isolation clothing (9). It was highlighted that the corresponding protective equipment should be removed in accordance with the standard process when leaving the polluted area and potentially polluted areas, and, as far as possible, pollution should not happen during the removal. Since the wearing of protective equipment impacts the operation sensitivity and flexibility of nursing staff, the wearing of three-level protective equipment will also impact the hearing, vision, and touch of nursing staff, resulting in poor communication between colleagues, poor operations, and even operation failure (10, 11). Thus, situation simulation training of wearing protective equipment should be performed in batches to lessen the negative impact of wearing protective equipment on nurses (Figure S1).

We outline recommendations for the PPE of the medical staff in anesthesia surgery centers for three-level screening (Table 1).

**Table 1:** Recommendations for the personal protective equipment (PPE) of the medical staff in anesthesia surgery centers.

**Protective level**	**Scope of application**	**PPE**	**Replacement time**
Level 1	Patients with no fever during elective operation	Work clothes, disposable work caps, and disposable surgical masks were to be worn. Latex gloves were also worn when in contact with blood, body fluids, secretions, or excreta, and goggles or protective face screen were worn when carrying out tracheal intubation, sputum aspiration, tracheal extubation, and other possible ways to generate aerosols.	Disposable surgical masks had to be changed every 4 h if they were not contaminated or wet. Disposable working caps and clothes had to be changed every 8 h and were to be replaced in time if contaminated or wet; goggles or protective screens had to be replaced following each operation.
Level 2	Patients with fever during elective operation	Work clothes, disposable work caps and disposable medical protective masks (N95 type masks and above), disposable protective clothing, disposable waterproof isolation clothes, waterproof shoe covers, double latex gloves, and goggles or protective face screens were worn.	Disposable medical protective masks had to be replaced every 4 h if not contaminated or wet. Disposable working caps and clothes had to be replaced every 8 h and had to be replaced in time if contaminated or wet; following each operation, the disposable waterproof protective clothing, disposable protective clothing, waterproof shoe covers, and goggles or protective face screen were to be replaced.
Level 3	Patients with suspected or confirmed COVID-19 during emergency surgery	Work clothes, disposable working cap and comprehensive respirator, disposable protective clothing, disposable waterproof isolation clothes, waterproof shoe covers, and double latex gloves were worn.	All equipment had to be replaced after each operation.

**Figure 3:**
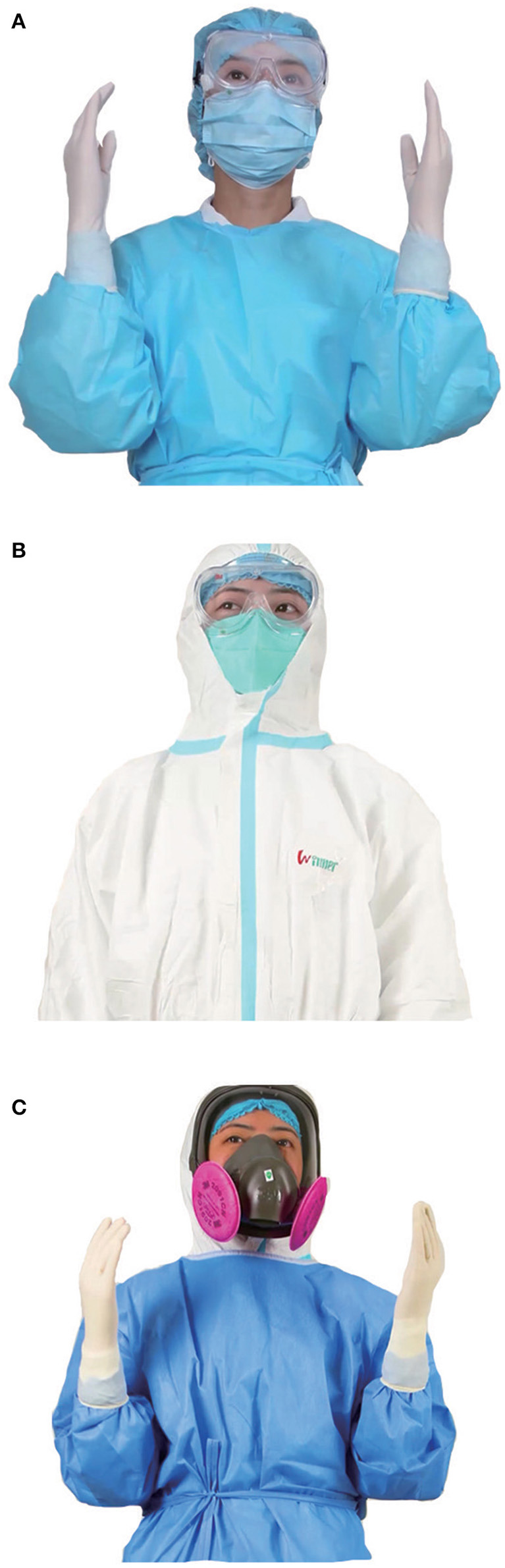
Personal protective equipment for medical staff at three levels. **(A)** Level 1 protection: for patients with no fever during elective operation. **(B)** Level 2 protection: for patients with fever during elective operation (wear disposable medical latex gloves with an outer layer). **(C)** Level 3 protection: for patients with suspected or confirmed COVID-19 during emergency surgery (wear disposable medical latex gloves with an outer layer).

For patients with no fever during selective operation, we carried out level 1 protection (Figure 3A): Work clothes, disposable work caps, and disposable surgical masks were to be worn. Latex gloves were also worn when in contact with blood, body fluids, secretions, or excreta, and goggles or a protective face screen were worn when carrying out tracheal intubation, sputum aspiration, tracheal extubation, and other possible ways to generate aerosols. For the replacement time, disposable surgical masks were to be changed every 4 h if they were not contaminated or wet. Disposable working caps and clothes had to be changed every 8 h and replaced in time if contaminated or wet; the goggles or protective screen had to be replaced following each operation.

For patients with fever during selective operation, we implemented level 2 protection (Figure 3B): Work clothes, disposable work caps, and disposable medical protective masks (N95 type masks and above), disposable protective clothing, disposable waterproof isolation clothes, waterproof shoe covers, double latex gloves, and goggles or a protective face screen were worn. In terms of replacement time, disposable medical protective masks had to be replaced every 4 h if not contaminated or wet. Disposable working caps and clothes had to be replaced every 8 h and were replaced in time if contaminated or wet; following each operation, the disposable waterproof protective clothing, disposable protective clothing, waterproof shoe cover, and goggles or protective face screen had to be replaced.

For patients with suspected or confirmed COVID-19 during emergency surgery, we carried out level 3 protection (Figure 3C): work clothes, disposable working cap, and comprehensive respirator, disposable protective clothing, disposable waterproof isolation clothes, waterproof shoe covers, and double latex gloves were worn. In terms of the replacement time, all equipment was replaced following each operation.

## Special Environmental and Human Resource Management

### Strict Environmental Management

Windows were opened to ventilate, the air conditioning was turned off, and the air was disinfected thrice a day in the anesthesia recovery room, the office area, and the dining room. Office desktops, mice, keyboards, printers, walkie talkies, computers, and other public facilities had to be wiped with 500 mg/L chlorine-containing disinfectant thrice a day. The door handles of public toilets, changing rooms, and duty rooms had to be disinfected every day, and toilet paper was provided outside the door for use as an anti-pollution measure. To avoid cluster-dining and cross-infection, the dining plan of the dining room of the anesthesia operation center was adjusted: self-service dining was canceled and modified to aid with taking a box meal; the dining time was controlled to about 20 min in batches and to a limited number of people, and no chatting was allowed during the dining period; most of the dining chairs were removed to ensure that the distance between each diner was not <1 m.

### Human Resource Management Under Special Situations

We listed details on working shift durations for various employees. Recently, research has indicated that we should consider minimizing staff exposure to COVID-19 patients by optimizing work shifts (12). The numbers of open operating rooms, anesthesiologists, and anesthesia nurses who needed to work were determined in accordance with the amount of surgery on the next day. The working time of medical staff was around 8–10 h with rest breaks. The anesthesiologists in the surgery room were divided into three batches with three different shifts, 8:00–16:00, 9:00–17:00, and 16:00–19:00; the anesthesiologists in PACU were split into two batches with three shifts, 7:00–17:30 and 9:00–19:00; the nurses and transport workers were split into four batches with four shifts: 8:00–16:00, 9:00–17:00, 10:00–18:00, and 11:00–19:00. These schedules ensured the smooth operation and lessened staff gathering. Cleaning personnel were split into two batches with two shifts, 7:00–15:00 and 15:00–23:00, for two-liner change.

## Online Teaching Management

Our hospital is the teaching hospital of a large medical center. Because of the impact of the epidemic situation and the delay of the students' school opening time, we investigated new teaching methods and conducted live broadcasts, question answering, and discussions of theoretical courses via various networked teaching or conferencing platforms. To avoid the gathering of personnel, on-site teaching was canceled and modified to record teaching videos for the Wechat group, asking and answering questions from the group. After 3 days of file sharing, online tests related to the teaching content were taken to test the learning effect and ensure the teaching quality. During the COVID-19 epidemic, we should strengthen the training of interns on the knowledge and skills related to epidemic prevention and control and pay special attention to training on the awareness, skills, and psychological adjustment of prevention and control. During the outbreak of COVID-19, interns did not carry out invasive operations so as to lessen the possibility of occupational exposure.

## Discussion

Under the COVID-19 pandemic, the ways to ensure the gradual recovery of anesthesia nursing unit and avoid cross-infection of the anesthesia nursing unit in a West China hospital can be outlined according to six aspects.

1. Hospital and operating room channel management: The hospital and the nursing department should adopt a reasonable layout of the medical space, optimize the treatment process and patient transfer process, implement “three-channel management,” and build a physical barrier.
2. Three-level screening management of patients and medical staff: Patients and medical staff should perform epidemiological history screening and be under the control of three-level screening management to implement multiple filtering and cut off the source of infection.
3. Classification management of patients undergoing anesthesia and recovery: Ordinary patients and suspected/confirmed patients with COVID-19 should be managed in accordance with anesthesia and operation classification and precautions to ensure the safety of patients and staff.
4. Training management of medical personnel: Training should be given to medical staff on COVID-19 prevention and control to improve personal protection ability, particularly covering medical staff nursing behavior, selection of protective equipment, and specification of the wearing and taking off process based on “three-level protection” under different situations.
5. Special environmental and human resource management: The strict management of the environment of the department should be strengthened, aggregation lessened, and the supply of ppe ensured; flexible human resource management can ensure the smooth completion of daily work while reducing the number of medical staff and exposure as much as possible;
6. Online teaching management: On-site teaching should be replaced with online teaching to ensure the safety of students and to complete the teaching plan.

Some other groups have also shared their clinical experiences of managing patients under COVID-19. Sorbello et al. (13) described key elements of clinical management in Italy, including safe oxygen therapy, airway management, PPE, and non-technical aspects of caring for patients diagnosed with COVID 2019. In these settings, there are specific factors that must be highlighted: oxygen administration and non-invasive ventilation of spontaneously ventilating patients; airway management of patients requiring tracheal intubation; clinical management with PPE; and human factors. Dexter et al. (14) suggested an evidence-based approach for the optimization of infection control and operating room management to defend perioperative COVID-19. The approach included improved hand hygiene, environmental cleaning via surface disinfectants and ultraviolet light, improved vascular care, patient decolonization, and surveillance optimization, which was in part consistent with our strategies. Recently research indicated that a combination of effective patient testing strategies, intelligent work planning, and thoughtful resource management could optimize treatment capacity, limit healthcare worker exposure, limit unnecessary use of PPE, and ensure high-quality patient care while avoiding staff overexertion (12, 15).

Through the implementation of the previously mentioned epidemic prevention and control strategies, anesthesia nursing work in our department is performed in an orderly and safe manner. Theoretical teaching is arranged according to the plan, but online teaching and discussion are more popular with students. These epidemic prevention and control strategies are based on China's national conditions, local epidemic situation, and hospital conditions, so anesthesia nursing colleagues can select from them in accordance with their own specific conditions.

## Author Contributions

PZ, RZ, LYe, and TZ contributed to the conception and design of the study. LYi, XY, YM, HW, LYe, and TZ contributed to the acquisition, analysis, and interpretation of data. All authors were involved in the revision of the manuscript, provided intellectual content of critical importance, and read and gave final approval of the version to be published.

## Conflict of Interest

The authors declare that the research was conducted in the absence of any commercial or financial relationships that could be construed as a potential conflict of interest.

## Supplementary Material

Figure S1 Training on protective equipment for medical staff in the operating room. **(A)** Level 1 protection training; **(B)** level 2 protection training.
