# Supplementary figures and images for: Emergency Response Measures for Anesthesia Nursing During the COVID-19 Pandemic: West China Hospital Experiences
Source: Front Med (Lausanne). 2020 Jul 22;7:460. doi: 10.3389/fmed.2020.00460 (PMC7387662; doi:10.3389/fmed.2020.00460)

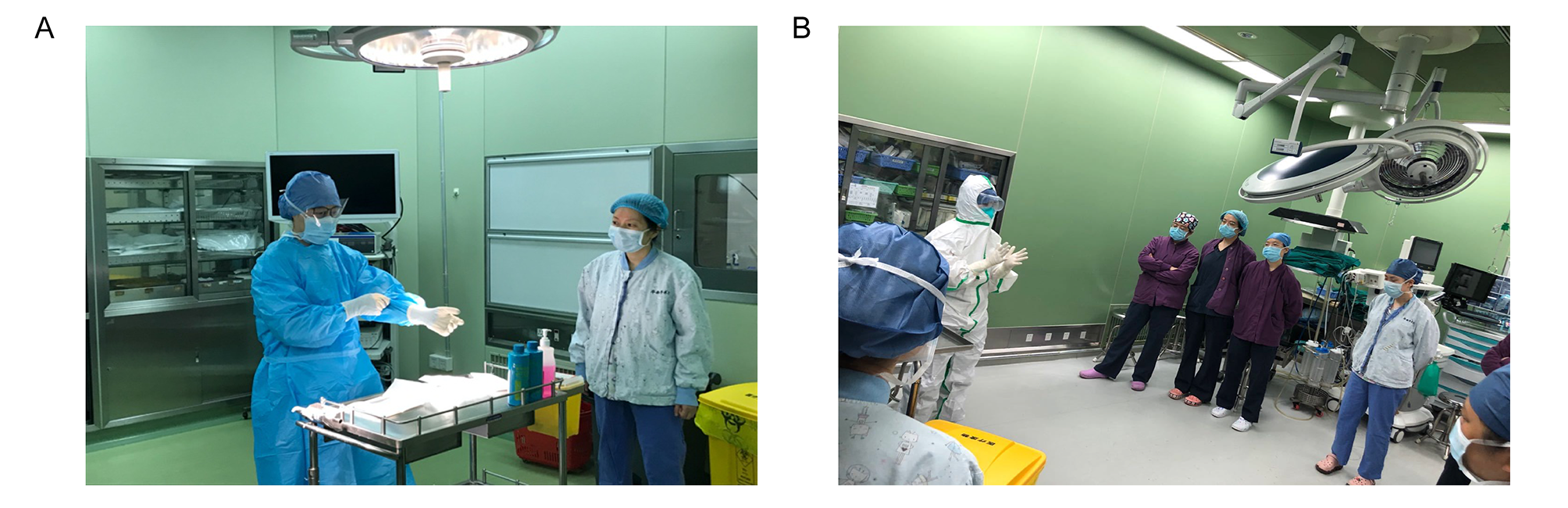

Supplement: Figure S1 — Training on protective equipment for medical staff in the operating room. (A) Level 1 protection training; (B) level 2 protection training. [file Image_1.TIF]
